# Spatial correlations and optical properties in three-dimensional deterministic aperiodic structures

**DOI:** 10.1038/srep13129

**Published:** 2015-08-13

**Authors:** Michael Renner, Georg von Freymann

**Affiliations:** 1 Department of Physics and Research Center OPTIMAS, University of Kaiserslautern, Erwin-Schrödinger-Str. 56. D-67663 Kaiserslautern, Germany; 2 Fraunhofer-Institute for Physical Measurement Techniques (IPM), Erwin-Schrödinger-Str. 56, D-67663 Kaiserslautern, Germany

## Abstract

Photonic systems have strongly varying optical properties depending on the spatial correlations present in a given realization. In photonic crystals the correlations are spatially periodic forming Bravais lattices whereas the building blocks of an amorphous medium are randomly distributed without any long-range order. In this manuscript we study the optical properties of so-called deterministic aperiodic structures which fill the gap between the aforementioned two limiting cases. Within this group we vary the spectrum of the spatial correlations from being pure-point over singularly-continuous to absolutely-continuous. The desired correlations are created in direct-laser written three-dimensional polymer structures using one construction principle which allows us to attribute the optical behaviour solely to the encoded spectrum. Infrared reflection measurements reveal the characteristic response of each spectral type verifying the successful fabrication of large deterministic aperiodic structures. To prove the presence of the correlations in all directions we perform transmission experiments parallel to the substrate by means of micro-optical mirrors placed next to the structures. Transport measurements reveal a strong dependence of the effective beam width at the output facet on the encoded lattice type. Finally, we reproduce the lattice type dependent transport behavior in numerical calculations ruling out extrinsic experimental reasons for these findings.

The purely mathematical studies of aperiodic tilings in the 1970s soon became a matter of practical importance when in 1984 Shechtmann *et al.* discovered a metal alloy having long-range order but lacking translational symmetry^1^,^2^. In the wake of this unexpected discovery Levine and Steinhardt^3^ introduced the concept of quasicrystals which marks the beginning of a new field in solid-state physics. Quasicrystals belong to the broader class of deterministic aperiodic (DA) systems considered intermediate between the crystalline and amorphous state. Generally, one can classify DA systems by its lattice spectrum of which there are three fundamental types given by Lebesgue’s decomposition theorem: pure-point spectra as in the case of all quasicrystals, furthermore singularly-continuous and absolutely-continuous spectra^4^. The latter spectral type also applies to completely random distributions. To unveil relations between these different kinds of aperiodic lattices and the resulting physical properties model Hamiltonians have been used in nearest-neighbour, tight-binding approximations^5^. Such theoretical studies found that aperiodic potentials derived from self-similar sequences of pure-point and singularly-continuous type have an energy spectrum of infinitely many gaps and zero bandwidth of allowed states. Thus, the energy spectrum itself is singularly-continuous. However, the nature of the energy spectrum for systems based on absolutely-continuous DA sequences is not fully resolved yet^6^. Besides theoretical investigations there are plenty of experimental studies concerning DA systems. In addition to experiments on electronic excitations^7^,^8^ the study of optical DA systems received increasing attention, mainly due to absence of interfering electron-electron interactions^9^. One-dimensional systems in the form of multilayer stacks are the most frequent experimental realization^10^,^11^ followed by systems with aperiodic order extending across a plane^12^,^13^. Fabricating three-dimensional DA systems is a very challenging task. Nevertheless, quasicrystals of relatively small thickness have been fabricated using stereolithography, optical interference holography and direct laser writing (DLW)^14^,^15^,^16^. Technological advances in DLW have enabled samples of increasing thickness as shown in a recent study on large-sized DA structures with pure-point and absolutely-continuous lattice spectra^17^. Here, we expand on this work by including structures of singularly-continuous type in order to present a comprehensive study of the optical properties of DA photonic systems. Additionally, we investigate the question how structures containing correlations drawn from a stochastic process compare to structures derived from the Rudin-Shapiro sequence as both share an absolutely-continuous spectrum but have different topological entropy^4^.

Lattices of all three possible spectral types can be conveniently generated by means of substitution sequences. Iteratively applying the rule *ω*_FI_:A→AB, B→A starting from the letter A one obtains the Fibonacci sequence with a pure-point spectrum whereas the rule *ω*_TM_:A→AB, B→BA leads to the singularly-continuous Thue-Morse sequence. Using *ω*_RS_:AA→AAAB, AB→AABA, BA→BBAB, BB→BBBA produces the Rudin-Shapiro sequence which has an absolutely-continuous spectrum^18^. To assemble a three-dimensional entity from these sequences we start from a simple cubic crystal. We successively assign the letters of the chosen sequence to the intervals of the (100) planes and change all intervals being labelled A to a certain value *l*_A_ and all intervals labelled B to *l*_B_. The same modifications are made to the (010) as well as the (001) planes. We choose the ratio of the two different lengths to equal the golden mean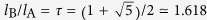. This choice leads to the canonical Fibonacci structure having the additional property of self-similarity^19^.

## Experimental section

**Figure 1:**
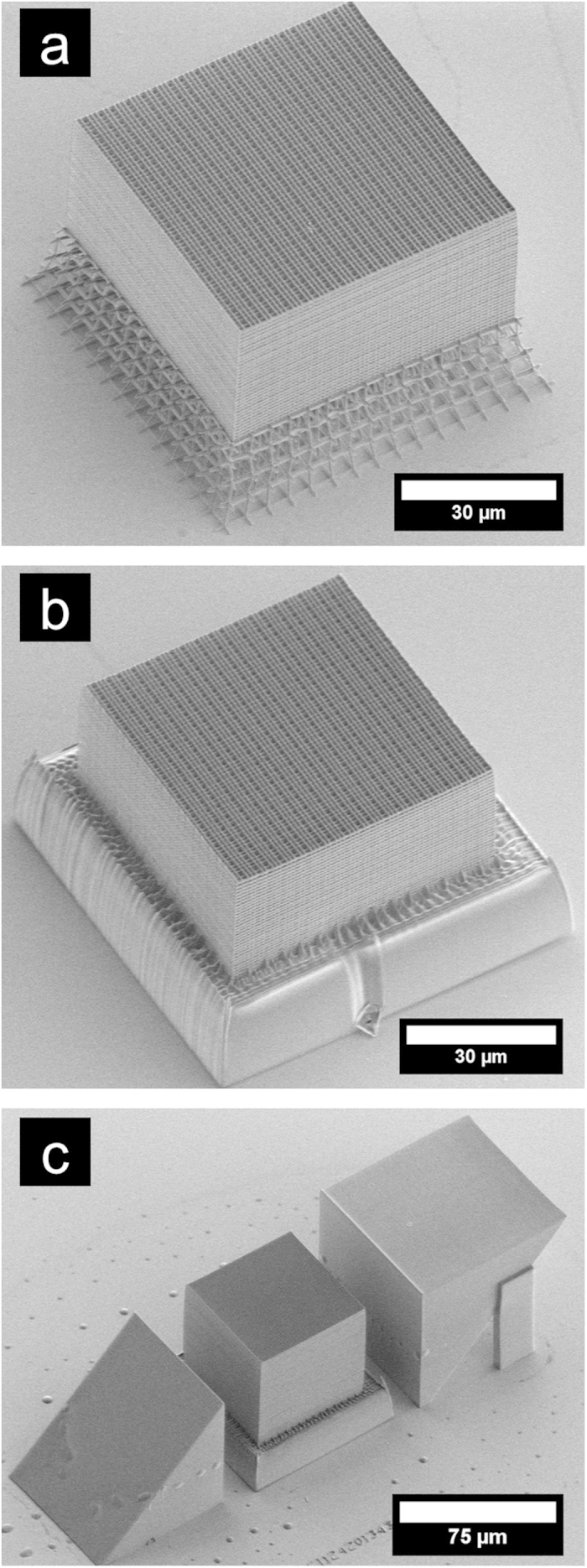
Deterministic aperiodic structures. (**a**) Scanning electron micrograph of a 30 μm thick 3D Fibonacci tiling fabricated on top of a base to release shrinkage induced strain. (**b**) Same structures as (**a**) after filling of the base allowing well-defined excitation in transmission experiments. (**c**) Structure in between micro optical mirrors for excitation in the direction parallel to the substrate. The side lengths of the half cubes are 70 μm and 80 μm for the bottom-to-side and the side-to-top mirrors, respectively.

The three-dimensional DA structures are realized by solidifying all aperiodically arranged planes {100} in a negative-tone liquid resist using a commercial direct laser writing system (IP-DIP and Photonic Professional, Nanoscribe GmbH). The written planes contain intended perforations allowing the developer to wash away the non-solidified resist^17^. Hence, the resulting refractive index contrast is that of polymer (n = 1.55) to air. With *l*_A_ = 0.84 μm and *l*_B_ = *l*_A_·τ ≃ 1.359 μm we choose the smallest possible values for our lithographic system in order to shift the spectral response to shorter wavelengths. All structures under investigation have the same footprint of about 70 μm and are fabricated on top of a base allowing for uniform shrinkage of the polymer during the development step (Fig. 1).

**Figure 2:**
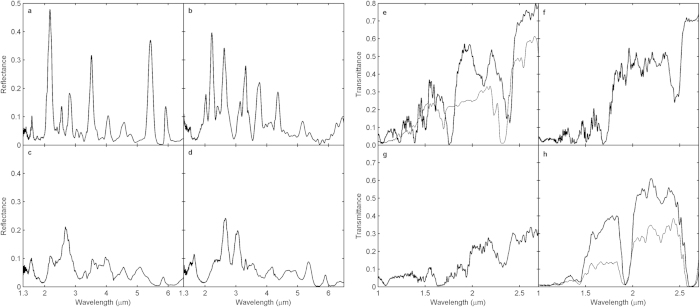
Reflectance spectra. (**a**–**d**) Fibonacci (**a**), Thue-Morse (**b**), Rudin-Shapiro (**c**) and a random (**d**) tiling with height h ≃ 72 μm (about 60 elements along the directions parallel and 80 elements perpendicular to the substrate). Transmittance spectra. (**e**–**h**) Parallel (thick) and perpendicular (faint) to the substrate of a Fibonacci structure (**e**). Parallel to the substrate for a Thue-Morse (**f**) and Rudin-Shapiro structure (**g**). Perpendicular to the substrate before (faint) and after (thick) filling of the base (**h**). The transmittance spectra are restricted to the non-absorptive region of the polymer.

One direct way of comparing the optical properties of different lattice types is to measure the reflectance from the DA structures. The measurements are performed with short- and mid-wavelength infrared light using a Fourier-transform infrared spectrometer (FTIR, Bruker Vertex 70v). To avoid spectral smoothing by angle-averaging the numerical aperture of the Cassegrain objective is reduced to an half opening angle of about 7.5° using a normal incidence geometry. The results for structures of identical thickness *d* = 73 μm are shown in Fig. 2 a–d. The most noticeable difference can be observed between the reflectance from Fibonacci and Rudin-Shapiro structures. While the former has a series of prominent peaks with up to 50% reflectance, the latter shows a homogenous response over the whole spectral range with only minor peaks – a behavior which is shared with the structure containing random correlations. This similarity is not unexpected since both have an absolutely-continuous lattice spectrum. In contrast, the spectral response of the Fibonacci structure is already fully developed showing strong reflectance form a set of hierarchically arranged pseudo stop bands which are related to the most significant coefficients in the quasi crystalline structure factor^18^. The spectral positions and the relative heights of the peaks at longer wavelengths than the fundamental reflection (*λ* = 2.2 μm) can be well described by 1-dimensional scattering matrix calculations of a multilayer stack (not shown). At shorter wavelengths this model fails indicating increasing scattering out of the initial propagation direction. The reflectance from the singularly-continuous Thue-Morse structure can be characterized as being intermediate between the reflectance from the Fibonacci and Rudin-Shapiro structure. It still contains peaks of reasonable strength as in the case of the Fibonacci structure but these appear on a relatively broad background. Generally, the fabricated structures show some polymeric absorption in the investigated spectral range. However, the main absorption regions around *λ* = 2.83 μm and *λ* = 3.38 μm have no or little influence on the reflectance spectra.

To prove that the DA structures are truly three-dimensional we measure the transmittance along two orthogonal directions within our samples. The direction parallel to the substrate is accessed by means of micro-optical mirrors fabricated next to the DA structures in a single direct laser writing step (see Fig. 1c). The micro-optical mirrors consist of cubes cut into halves along a surface diagonal and rely on total internal reflection (TIR). For our illumination geometry with an half opening angle of 7.5° more than 90% of the incoming light is below the critical angle (*θ*_*c*_ = 40.2°). Any remaining spectral features of the micro-optics are removed from the obtained transmittance spectra by referencing against a pair of empty mirrors.

First, we compare the transmittance of a Fibonacci structure along two perpendicular directions. The spectrum obtained normal and parallel (Fig. 2e) to the substrate shows pronounced dips down to zero transmittance and a similar overall trend. However, the spectral positions of the main gaps of the transmittance normal to the substrate appear blue-shifted by approximately 70 nm and the spectral features below 2 μm are clearly smoothed out compared to spectrum obtained parallel to the substrate. These differences in the spectra are likely caused by the anisotropy of the structure itself. Due to the ellipsoidal “writing pen” oft the DLW system the two directions normal and parallel to the substrate are no longer equivalent in terms of an average refractive index. While the direction parallel to the substrate is now accessible by the micro-optics the normal direction is partly obstructed by the base used to compensate for shrinkage effects (see Fig. 1a). To rule out that this base leads to the observed smoothing of spectral feature at shorter wavelengths we perform a second DLW step. After immersing the structure in resist (IP-L 780, Nanoscribe GmbH) we convert the base into a solid polymer block by writing through the substrate with an air objective (NA = 0.5). As can be seen in Fig. 1b this second DLW step does not impair the structural quality. Figure 2h reveals that overall transmittance level is increased considerably after filling of the base, yet no sharper spectral features appear. Hence, the smoothed transmittance curve below 2 μm also results from the anisotropy of the structure.

Next, we compare the transmittance parallel to the substrate of a Fibonacci, Thue-Morse and Rudin-Shapiro structure (Fig. 2e–f). Again, the differences are most prominent between the transmittance of the Fibonacci and the Rudin-Shapiro structure. Concerning the absolute transmittance levels the former reaches 80% while the latter hardly exceeds 30%. In correspondence with the results in reflection and its absolutely-continuous lattice spectrum, the Rudin-Shapiro structure has only minor variations in the considered spectral range disregarding the gradually increasing transmittance to longer wavelengths. In contrast, we observe the fingerprints of a pure-point lattice spectrum in the transmittance of the Fibonacci structure. Several pseudo stop bands are visible and particularly around *λ* = 1.5 μm we find remarkably sharp features with transmittance variations of more than a factor of 2 within 15 nm small intervals. Finally, the transmittance of a Thue-Morse structure can be described as more fluctuating than Rudin-Shapiro but still smoother than Fibonacci in term of peak-to-valley modulation depth. Thus, this behavior nicely agrees with the results obtained in reflection.

**Figure 3:**
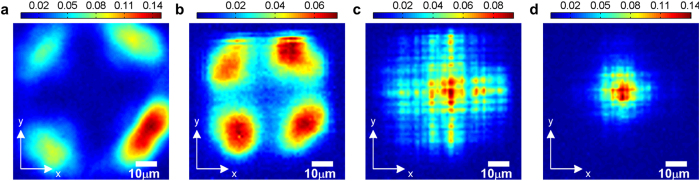
Measured transmission patterns for different modulation strengths. (**a**) Pattern obtained when no sample is present. The Cassegrain objectives are positioned such that the excitation (collection) focus is placed at the bottom (top) of the structures when introduced into the light path. (**b**) Periodic structure with equal elements. (**c**,**d**) Weakly modulated (±10%) (**c**) and full 1/τ (±23.65%) modulated (**d**) Rudin-Shapiro structure. Structure height is 60 μm. Patterns are shown at a wavelength of λ = 1.7 μm.

Adding the reflectance and transmittance of Fig. 2 leads to a value that is far from unity. This can be partly attributed to finite numerical aperture of the collection optics. However, most of the missing light is lost to the sides of the structure due to scattering events. The mean distance a photon travels before being scattered out of the structure is determined from thickness dependent transmittance measurements. Once coupled into the structure the scattering loss during propagation at λ = 1.7 μm is smallest for Fibonacci with a mean free path of *l*_FI_ = 236 μm followed by Thue-Morse, random and Rudin-Shapiro (*l*_TM_ = 103 μm, *l*_Rand_ = 73 μm and *l*_RS_ = 67 μm, see Supplementary Fig. S1). In order to elucidate the propagation inside the DA structures we spatially resolve the transmitted light by imaging the exit facet onto a focal plane array (FPA) attached to the FTIR. For excitation we use light from a tungsten halogen lamp which is focused to a circular spot of 16 μm diameter and centered on the input facet of the structure. In a first experiment, we study the influence of the modulation strength given by the ratio of the two length elements. To this end, we fabricate a set of Rudin-Shapiro structures of equal size but varying ratio *l*_B_/*l*_A_. For *l*_A_ = *l*_B_ = 1.1 μm, hence for zero modulation strength, we obtain a simple cubic photonic crystal having only marginal influence on the transmitted light distribution (Fig. 3b). And indeed, removing the structure from the light path, we observe the same four-fold symmetric distribution which is slightly broadened due to the lower refractive index of air (Fig. 3a). Increasing the modulation strength to ±10% (*l*_A_ = 0.99 μm, *l*_B_ = 1.21 μm) results in a pattern covering substantial parts of the sample surface (Fig. 3c). In contrast, for the canonical ratio (*l*_A_ = 0.84 μm, *l*_B_ = *l*_A_ · τ ≃ 1.359 μm corresponding ±23.65% modulation) the transmitted light is primarily confined to the point of excitation. Interestingly, the peak transmission in the center of the structure is the highest for the strongest modulation which indicates that the modes become more localized with increasing modulation.

**Figure 4:**
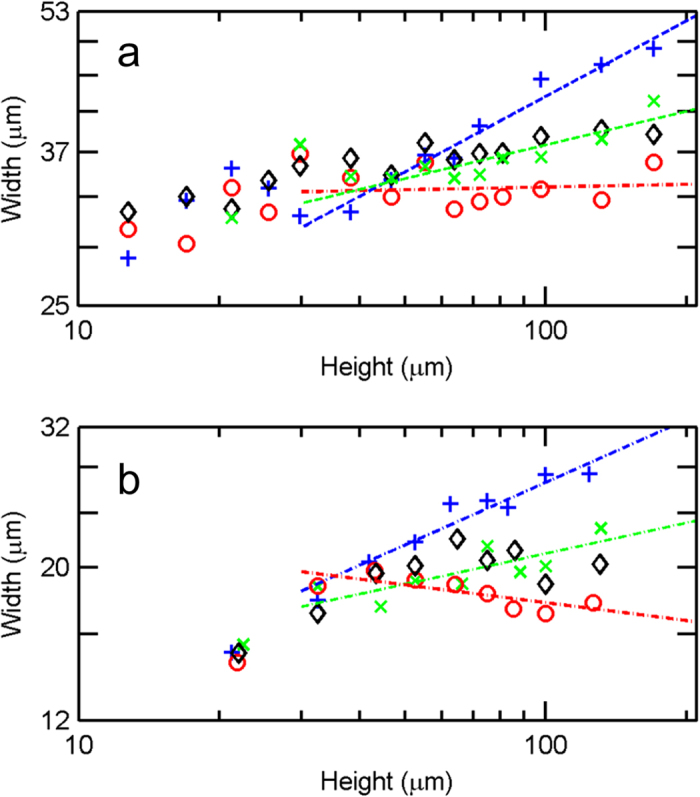
Measured and calculated effective widths. (**a**) Measured effective width vs. height for Fibonacci (+), Thue-Morse (×), random (◊) and Rudin-Shapiro (o) structures at λ = 1.7 μm shown on a double-logarithmic scale. Different trends can only be observed for thick structures (>50 μm). (**b**) Double-logarithmic plot of calculated effective width vs. height for a Fibonacci (+), Thue-Morse (×), random (◊) and Rudin-Shapiro (o) structure at the same wavelength as in (**a**).

**Figure 5:**
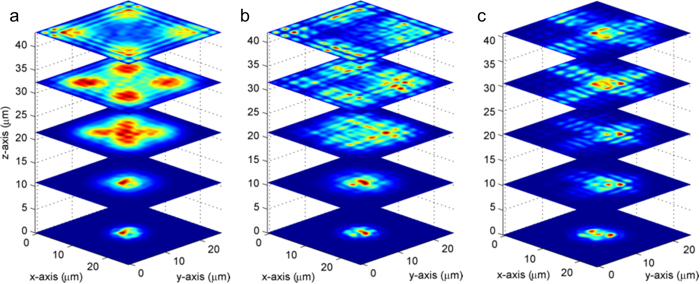
Calculated transmission patterns. (**a**) Transmission pattern of periodic structure with equal elements. (**b**,**c**) Weakly modulated (±10%) (**b**) and full 1/τ (±23.65%) modulated (**c**) Rudin-Shapiro structure. Patterns are obtained from 20 individual FDTD simulations runs (λ = 1.7 μm).

In a next step, we investigate the observed mode localization by measuring the effective width of the transmitted beam at different propagation distances for fixed modulation *l*_B_/*l*_A_ = *τ*. As we cannot extract the light propagation within a single structure we fabricate a set of structures with increasing thickness from 13 μm up to 170 μm. After recording the light distribution *I*(*x*, *y*) for the same excitation conditions as before we calculate the inverse participation 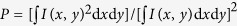 to finally extract an effective width *ω*_eff_ = *P*^−1/2^. Figure 4a shows the obtained width vs. propagation distances *z* on a double-logarithmic scale. Below a structure height of about 50 μm no differences between the lattice types can be discerned. Above this height, however, we find noticeably different trends. While the effective width for Rudin-Shapiro structures levels at around *ω*_eff_ = 33 μm we find monotonically increasing values for Fibonacci structures. From linear fits in Fig. 5a we can derive the exponent of a power-law relation *ω*_eff_ = *z*^*v*^ in order to quantitatively compare the different lattice types. The smallest exponent is found for the Rudin-Shapiro structure, followed by the structures containing random and Thue-Morse correlations(*v*_RS_ = 0.01 ± 0.032 < *v*_Rand_ = 0.06 ± 0.016 < *v*_TM_ = 0.12 ± 0.028. The error interval equals one standard deviation). The largest exponent is obtained for the structure based on the Fibonacci sequence (*v*_FI_ = 0.28 ± 0.021). These findings might illustrate the consequences of different eigenmodes associated with each lattice type. The light distribution found in absolutely-continuous structures is confined to the smallest area indicating exponentially localized eigenmodes^17^. As can be seen from the derived exponents Rudin-Shapiro could be advantageous over random correlations when localized modes are needed, e.g., for random lasers. At this point we would like to emphasize that the localization we refer to takes place in the plane perpendicular to the propagation direction. In time-resolved transmission experiments we do not observe any largely enhanced photon dwell times inside the structures, even at shorter wavelengths (see Supplementary Fig. S2).

### Numerical section

The intricate sample fabrication via DLW inevitably leads to some deviations from the ideal structure due to polymer shrinkage or residual surface roughness. To separate these extrinsic effects from the properties of an ideal sample and to eliminate some experimental limitations we perform three-dimensional numerical simulations using a commercial finite-difference time-domain package (Lumerical Solutions, Inc.). Due to limited memory (64 Gb) large-sized structures can be calculated only for a narrow spectral range and a reduced footprint. That is why we concentrate on qualitatively reproducing the results found for the effective widths at a single wavelength. To account for the incoherent excitation in the experiment we run a number of individual simulations using the point spread function (PSF) of a Cassegrain objective as the source (NA = 0.5, excitation angles 15°–30°). From run to run the polarization and position of the PSF is varied randomly within a circular area of 5 μm diameter. Inside the structure monitors record the field distributions at several positions along the propagation direction for a fixed wavelength. The recorded fields are projected to the far-field in the forward direction followed by the removal of all components lying outside of the acceptance cone of the objective used to collect the light in transmission (same Cassegrain objective as for excitation). After reversing the far-field projection the incoherent sum of all simulation runs is calculated. Due to this procedure the resulting field distributions are directly comparable to the experimental results.

To demonstrate the validity of this approach we numerically calculate the behavior found for increasing modulation strength in a Rudin-Shapiro structure. And indeed, for zero modulation, we obtain a four-fold symmetric pattern similar to the experimental result (see Fig. 5a for z  = 32 μm and Fig. 3b for comparison). For longer propagation (z > 30 μm) the light starts to interact with the lateral boundaries of the structure. The effect of increasing modulation strength is also found to be in good agreement with the experiment. While a modulation of ±10% leads to a light distribution which is spread across the whole plane, simulations for the canonical modulation (±23.65%) yield a mode pattern which is localized at the point of excitation (Fig. 5b,c and 3c,d for comparison). By changing the simulation to normal Gaussian PSF for excitation and collection we exclude the peculiar transfer function of the Cassegrain objectives to have any effect on the observed behavior.

For the comparison of the effective beam width in different lattice types the memory capacity limits the structures size to 33 × 33 × 130 μm^3^ at 68 nm mesh resolution. Convergence tests show that the extracted effective widths are converged to within 1% at this resolution. The results from the simulations displayed in Fig. 4b qualitatively reproduce the trends found in the experiment. The broadening of the beam is fastest in Fibonacci, followed by Thue-Morse and random structures (*v*_FI_ = 0.30 ± 0.036 > *v*_TM_ = 0.15 ± 0.044 > *v*_Rand_ = 0.07 ± 0.056). The exponents *v* nicely match the experimental values. Again, the slowest broadening is found for the Rudin-Shapiro structure. However, the exponent from the fit is negative indicating that the pattern even reduces in size during propagation. This effect might arise from the continuing extinction of modes being in close contact with the boundaries. As the lateral footprint of the calculated structures has only about half the size of the experimental structures this loss mechanism is more significant in the numerical calculations leading to the observed deviations from the experiment.

To summarize, we successfully fabricated three-dimensional DA structure of all three possible spectral lattice types and performed a study of the optical properties in the infrared spectral range. We have found characteristic fingerprints of the aperiodic lattices in both reflectance and transmittance spectra allowing a clear discrimination between the different lattice types. Furthermore, investigations of light transport inside DA structures revealed differences in the excited eigenmodes evolving differently with the propagation distance. Eigenmodes in Rudin-Shapiro structures are localized most strongly compared to other lattice types. These experimental findings were corroborated by numerical FDTD studies giving qualitatively similar results. Choosing a certain lattice type to obtain predictable eigenmodes properties presents a viable tool to enhance the functionality of many photonic systems, e.g., in the context of random lasing or light harvesting for energy purposes.

## Methods

Samples are fabricated with a commercial direct laser writing system (Photonics Professional, Nanoscribe GmbH) at a speed of 200 μm/s using a microscope lens of NA = 1.3 (100x) by Carl Zeiss. The negative-tone photoresist (IP-DIP by Nanoscribe GmbH, n = 1.52) is developed successively in PGMEA and isopropanol for 10 minutes each.

We measure infrared reflectance and transmittance spectra with a Fourier-transform spectrometer (Bruker Vertex 70v, middle-infrared globar source) combined with an infrared microscope (Bruker Hyperion 3000, 36x Cassegrain objective, liquid-N_2_-cooled MCT detector) using a circular light spot of 16 μm diameter at a resolution of Δν = 8 cm^−1^ wavenumbers. We tilt the sample and cover 3 quadrants of the objective to obtain a normal incidence geometry with a half opening angle of 7.5°. The reflectance spectra are normalized to a gold mirror. For measurements using the liquid-N_2_-cooled FPA detector (64 × 64 pixels) we employ a 36x Cassegrain objective (NA = 0.5, opening angles between 15°–30°) and a near-infrared halogen lamp. The pixel resolution of 1.1 μm leads to diffraction-limited intensity maps over the full spectral range. For a clearer visual representation the maps are resized by a factor of 4 using the MATLAB function *imresize* with bicubic interpolation. Spatially resolved transmittance spectra are collected at a resolution of Δν = 16 cm^−1^ with 1024 scans and are normalized to the glass substrate.

## Additional Information

**How to cite this article**: Renner, M. and von Freymann, G. Spatial correlations and optical properties in three-dimensional deterministic aperiodic structures. *Sci. Rep.* **5**, 13129; doi: 10.1038/srep13129 (2015).

## Supplementary Material

Supplementary Information
