# Supplementary material for: Spatial correlations and optical properties in three-dimensional deterministic aperiodic structures
Source: Sci Rep. 2015 Aug 13;5:13129. doi: 10.1038/srep13129 (PMC4534760; doi:10.1038/srep13129)
Supplement: Supplementary Information [file srep13129-s1.pdf]

Spatial correlations and optical properties in three-dimensional deterministic aperiodic structures

Michael Renner and Georg von Freymann*

Supplementary Information

Thickness dependent transmittance measurements

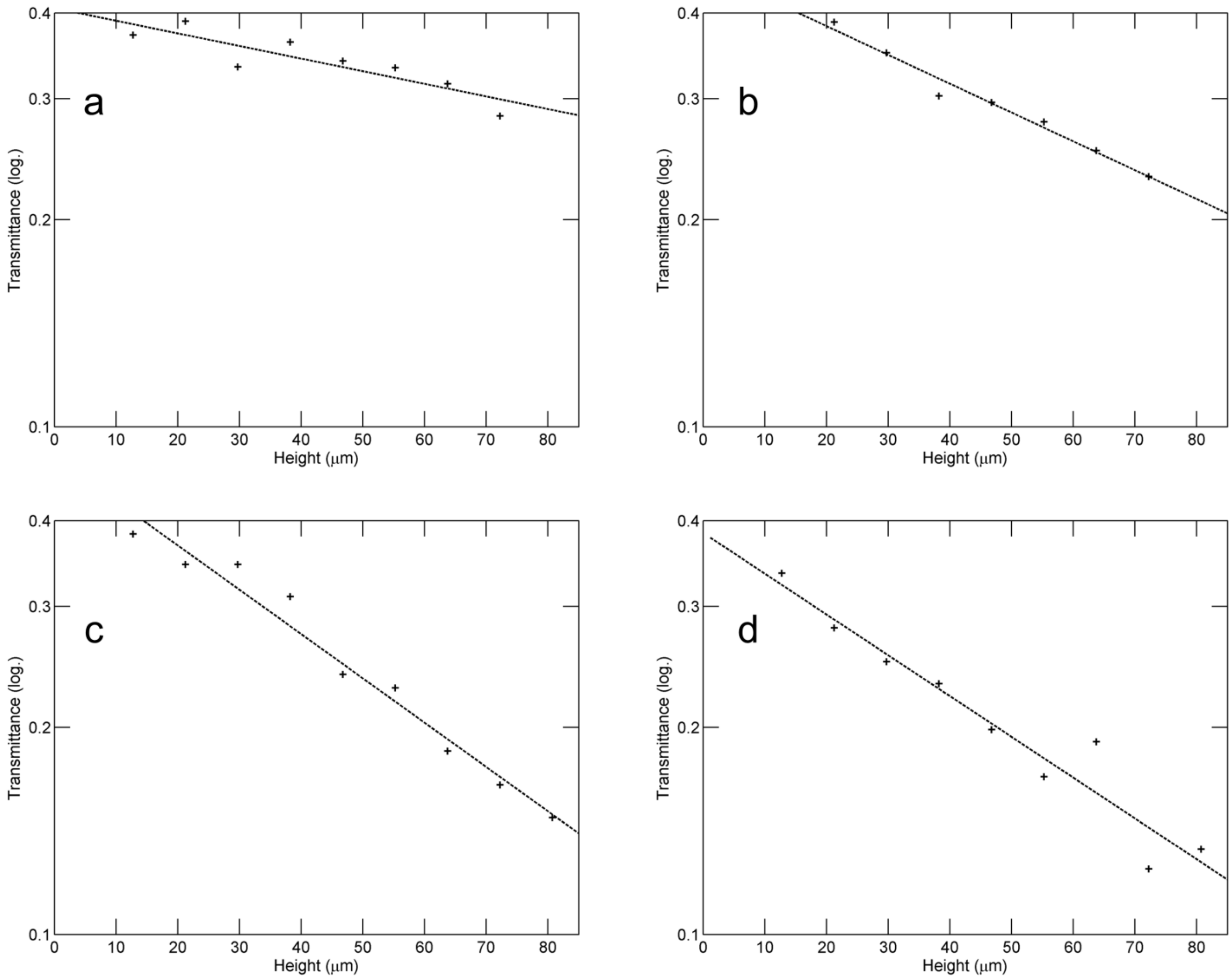

Supplementary Figure S1: Transmittance as a function of sample thickness for Fibonacci (**a**), Thue-Morse (**b**), Rudin-Shapiro (**c**) and random (**d**) structures at a wavelength of $\lambda = 1.7\ \mu m$ (±23.65 % modulation strength). FTIR measurements are performed in standard geometry (non-normal incidence) using a 36x Cassegrain objective. The excitation spot has a diameter of $16.7\ \mu m$ while the transmitted light is collected over the full structure surface.

Time-resolved transmission

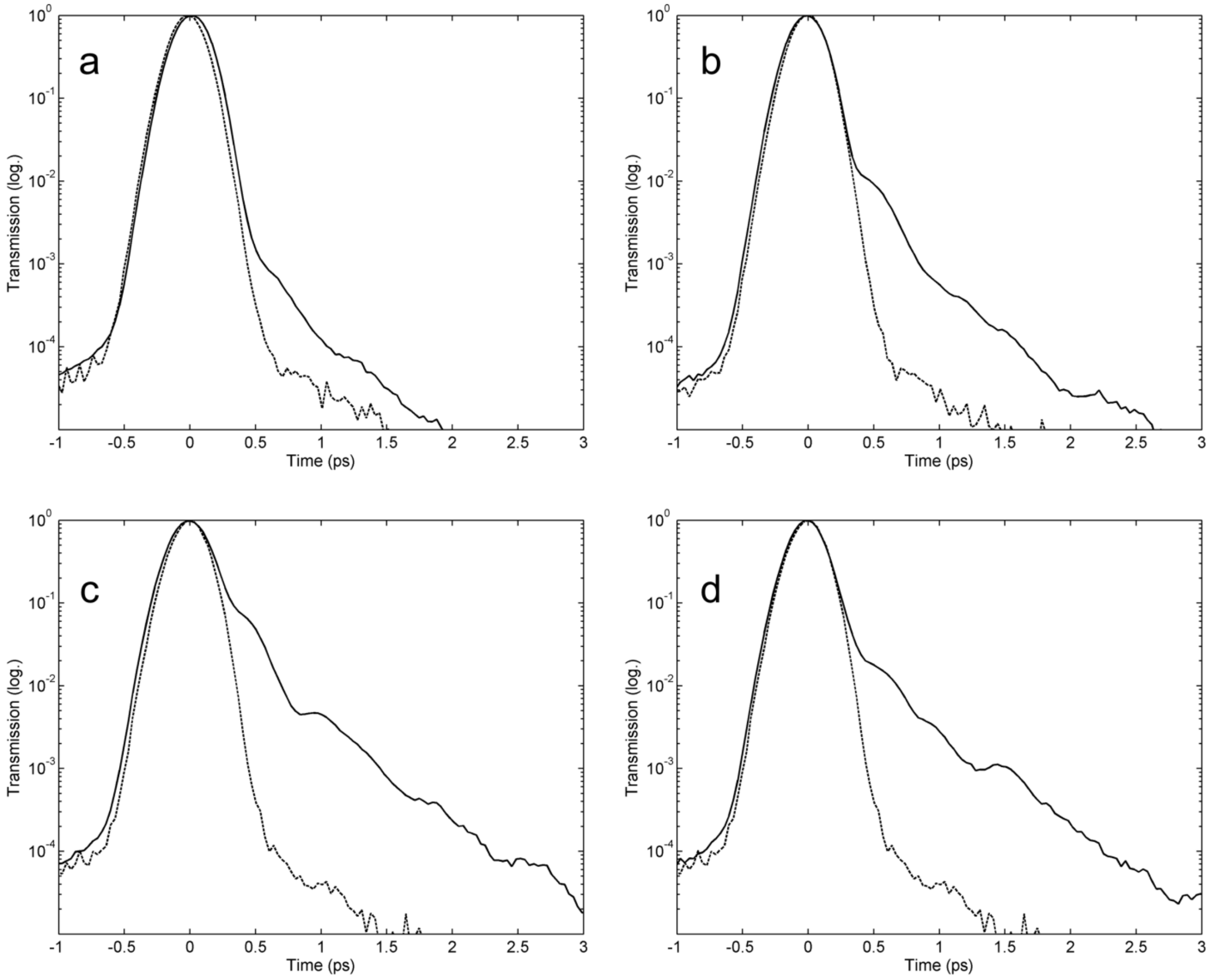

Supplementary Figure S2: Time-resolved transmission at a wavelength of $\lambda = 1.3\ \mu\mathrm{m}$ for a Fibonacci (**a**), Thue-Morse (**b**), Rudin-Shapiro (**c**) and random (**d**) structure of height $\mathrm{h} \simeq 72\ \mu\mathrm{m}$ (±23.65 % modulation strength). The reference trace with no sample present is drawn as a dashed line. The time-resolved transmission is measured using a nonlinear up-conversion technique. Transmitted photons having the same linear polarization as the input light are used for signal generation (parallel channel). The time-resolved transmission shown here is obtained from averaging 9 individual traces taken at slightly different sample positions.
